# Src family kinases interfere with dimerization of STAT5A through a phosphotyrosine-SH2 domain interaction

**DOI:** 10.1186/s12964-014-0081-7

**Published:** 2015-02-15

**Authors:** Dirk Fahrenkamp, Hildegard Schmitz-Van de Leur, Andrea Küster, Nicolas Chatain, Gerhard Müller-Newen

**Affiliations:** Institute of Biochemistry and Molecular Biology, Faculty of Medicine, RWTH Aachen University, Pauwelsstraße 30, 52074 Aachen, Germany; Department of Hematology, Oncology, Hemostaseology, and Stem Cell Transplantation, Faculty of Medicine, RWTH Aachen University, Aachen, Germany

**Keywords:** CML, STAT5, Src family kinases, Dimerization

## Abstract

**Background:**

Chronic myeloid leukemia (CML) is driven by the expression of the BCR-ABL oncoprotein. STAT5 is a BCR-ABL substrate and persistently activated by tyrosine phosphorylation in CML cells. Activated STAT5 (pSTAT5) drives proliferation and survival of leukemic cells and contributes to initial transformation and maintenance of the disease. In cytokine-induced STAT5 signaling, phosphorylation of STAT5A on Y694 leads to nuclear accumulation of the transcription factor, followed by DNA-binding and gene induction. However, Src-family kinases (SFK) mediate cytoplasmic retention of pSTAT5A leading to attenuated target gene expression and colony formation in CML cells.

**Results:**

In this study we show that autophosphorylation of Y416 in the highly conserved activation loop of SFK generates a potent recruitment site for the SH2 domain of STAT5A. Binding of the SH2 domain to the activation loop is required for STAT5A^Y694^ phosphorylation by SFK, but at the same time promotes the persistent cytoplasmic localization of the transcription factor as found in BCR-ABL^+^ leukemia. As a consequence of the complex formation between tyrosine-phosphorylated SFK and the SH2 domain of STAT5A, the dimerization of STAT5A is impaired. We further demonstrate that constitutively active STAT5A^S710F^ escapes from SFK-mediated cytoplasmic retention by enhancing STAT5A dimer stability.

**Conclusion:**

Our results reveal important structural aspects of cytoplasmic pSTAT5A found in myeloid leukemias and will contribute to the understanding of STAT5A mediated cytoplasmic signaling.

## Background

Signal transducer and activator of transcription (STAT) proteins are latent transcription factors that transmit signals from membrane-bound surface receptors to the nucleus. STAT proteins regulate proliferation, differentiation and apoptosis, involved in diverse biological processes such as hematopoiesis, tissue remodeling, immune responses and inflammation [1].

The STAT family is composed of seven members (STAT1, STAT2, STAT3, STAT4, STAT5A/B and STAT6) that share a structurally related and conserved domain architecture: The N-terminal multimerization domain, a coiled-coil domain, followed by the DNA-binding domain (DB), a linker domain (L), a single Src homology domain (SH2) and the transactivation domain (TA). In canonical Janus kinase (JAK)/STAT signaling, phosphorylated tyrosine residues in the cytoplasmic part of activated receptors serve as docking sites for SH2 domain containing proteins including STATs. Activation of STAT family members is mediated by the phosphorylation of a single conserved tyrosine residue, which results in dimerization via reciprocal phosphotyrosine-SH2 domain interactions, subsequent nuclear accumulation, DNA-binding and target gene expression [1].

STAT5 is of central importance for hematopoiesis in health and disease. Lymphocyte development is regulated by a variety of cytokines such as IL-2, IL-4, IL-7, IL-9, IL-15 and IL-21, which activate the JAK3/STAT5 signaling pathway through common γ chain (γ_c_) receptors [2]. JAK2 and SFK orchestrate STAT5 signaling events downstream of erythropoietin- (Epo), thrombopoietin- (Tpo) and epidermal growth factor (EGF) receptors [3,4].

Two highly related genes encode STAT5A and STAT5B proteins, which share 93% identity at the amino acid level with most of the differences located in the TA domain. STAT5A knockout mice exhibit a defective mammary gland development and impaired lactogenesis, whereas STAT5B expression is required for sexual dimorphism of body growth and liver gene expression [5,6]. Finally, gene targeting of both STAT5A and STAT5B in mice results in multiple hematopoietic defects involving the survival and proliferation of lymphoid lineages [7]. Previous results obtained from mice expressing N-terminally truncated forms of STAT5A/B additionally implicated STAT5 signaling in myeloid cell differentiation [8].

In contrast to physiological transient signaling, persistent STAT5 activation is a hallmark of many hematological malignancies including myeloproliferative neoplasms (MPNs) such as polycythemia vera (PV, JAK2^V617F^), essential thrombocythemia (ET, JAK2^V617F^), systemic mastocytosis (SM, cKIT^D816V^), acute myeloid leukemia (AML, FLT3-ITD) and chronic myeloid leukemia (CML, BCR-ABL) [3]. CML is a clonal myeloproliferative disorder, that is characterized by the presence of the Philadelphia chromosome t(9;22) leading to the expression of BCR-ABL, an oncogenic fusion protein with constitutive tyrosine kinase activity [9]. Despite the activation of numerous signaling pathways, STAT5 is mainly a BCR-ABL substrate and is persistently phosphorylated in CML cells [10]. Activated STAT5 drives proliferation and survival of leukemic cells and contributes to initial transformation and maintenance of the disease [7,11,12].

Mounting evidence suggests that STAT5A-mediated prosurvival signaling emerges from the cytoplasmic compartment, based on a cooperative crosstalk with the PI3-K/Akt signaling pathway. While mechanistic details of this crosstalk remain elusive, constitutively active STAT5A (STAT5A^S710F^) was found in a complex with Gab2, which promotes the cytoplasmic localization of the persistently phosphorylated transcription factor in myeloid leukemias [13].

In addition, several reports suggest a role of SFK such as Hck, Lyn and Fyn in mediating tyrosine kinase inhibitor (TKI) resistance in CML cells [14-16]. Moreover, Hck is frequently overexpressed in myeloid leukemias and links STAT5 to BCR-ABL signaling [17,18]. We recently showed that SFK promote the cytoplasmic localization of pSTAT5A in the presence of BCR-ABL through a mechanism that involves the SH2 domain of STAT5A. As a consequence, STAT5 mediated gene expression is permanently attenuated in CML cells [19]. However, a detailed understanding of the underlying mechanism is still lacking.

In this study, we provide evidence that the STAT5A SH2 domain tightly binds to the phosphorylated Y416 in the activation loop of SFK, resulting in the cytoplasmic localization and an impaired dimerization of the transcription factor. These processes are dominant over the BCR-ABL induced nuclear accumulation of STAT5A and therefore give a rational explanation for the cytoplasmic localization of STAT5A in a number of myeloid leukemias.

## Results

### Binding of STAT5A to SFK is mediated by the SH2 domain of STAT5A

**Figure 1:**
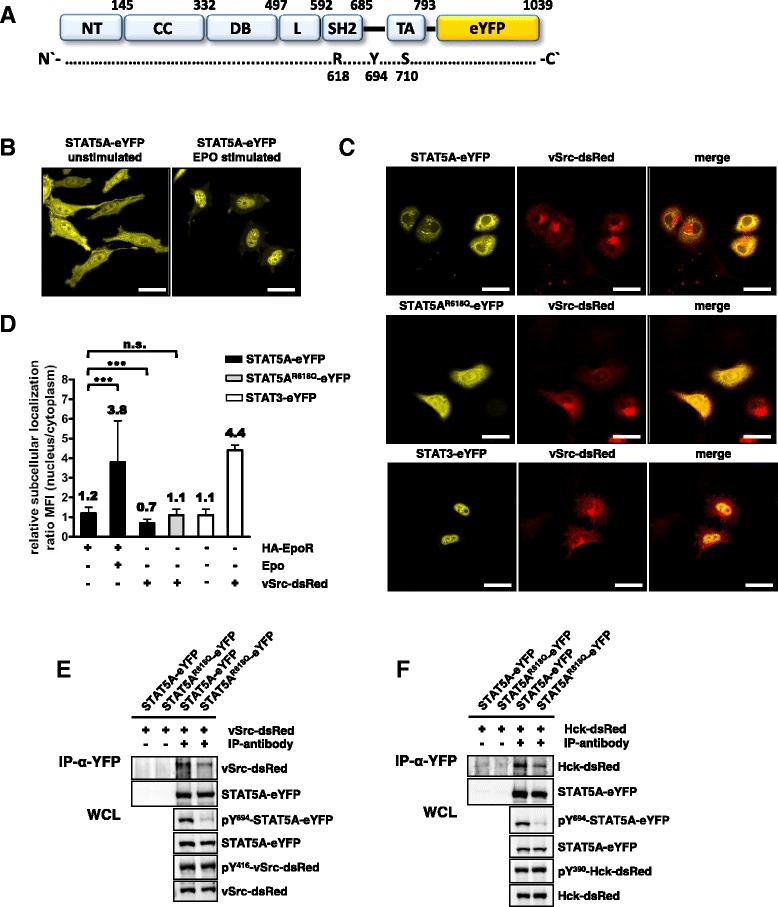
**The SH2 domain of STAT5A is required for efficient binding to Src kinases. (A)** Domain structure of fluorescently labeled STAT5A-eYFP. **(B)** Subcellular localization of STAT5A-eYFP in the absence or presence of Epo. HeLa T-REx HA-EpoR cells stably transfected with STAT5A-eYFP were stimulated with 1 U/ml Epo for 30 min and the localization of STAT5A-eYFP was analyzed by confocal microscopy. Scale bars: 20 μm. **(C)** Subcellular localization of STAT5A-eYFP (upper panel), STAT5A^R618Q^-eYFP (middle panel) and STAT3-eYFP (lower panel) was investigated in the presence of vSrc-dsRed. HeLa T-REx vSrc-dsRed cells were treated with 5 ng/ml doxycycline and transfected with the indicated constructs and the distribution of fluorescently labeled fusion proteins was analyzed after 24 h by confocal microscopy. Scale bars: 20 μm. **(D)** Quantification of the relative subcellular distribution of eYFP-labeled STAT3 and STAT5A constructs in HeLa T-REx HA-EpoR cells stably expressing STAT5A-eYFP **(B)** and HeLa T-REx vSrc-dsRed cells transfected with STAT5A-eYFP, STAT5A^R618Q^-eYFP or STAT3-eYFP **(C)**. The expression of the HA-EpoR and vSrc-dsRed was induced with 5 ng/ml doxycycline for 24 hours. Mean fluorescence intensities (MFI) of the cytoplasm and nucleus were determined using the Zen 2012 software and changes in the ratio between the compartments were plotted. The data shown are means ± SD of n = 30 cells and were statistically evaluated by Student’s *t*-test. ***p < 0.0005. n.s. = not significant. **(E + F)** HeLa T-REx FRT cells were co-transfected with plasmids coding for STAT5A-eYFP or STAT5A^R618Q^-eYFP and vSrc-dsRed or Hck-dsRed. Fluorescently labeled STAT5 was immunoprecipitated from cell lysates using a GFP antibody and analyzed by immunoblotting for the presence of vSrc-dsRed or Hck-dsRed 24 h after transfection. The expression and phosphorylation of STAT5A and vSrc/Hck proteins was analyzed in the whole cellular lysates (WCL) using antibodies against pY^416^-Src, Src, Hck, pY^694/699^-STAT5A/B and GFP.

In order to analyze STAT5A nucleocytoplasmic transport dynamics, we evaluated the subcellular distribution of fluorescently labeled STAT5A (Figure 1A) in an erythropoietin (Epo) responsive HeLa cell line. In the absence of a hormone stimulus, STAT5A-eYFP appeared almost equally distributed (nucleus/cytoplasm (n/c) ratio: 1.2, SD: 0.2, n = 30), whereas Epo treatment resulted in nuclear accumulation of the transcription factor (n/c ratio: 3.8, SD: 2.1, n = 30, p < 0.0001), demonstrating that STAT5A-eYFP imitates functional characteristics of untagged STAT5A (Figure 1B + D).

We have previously shown that SFK mediate the cytoplasmic localization of STAT5A in BCR-ABL expressing cells, a process that involves the SH2 domain of STAT5A [19]. To confirm these observations we analyzed the subcellular distribution of STAT5A-eYFP and STAT5A^R618Q^-eYFP, which harbors an inactivating mutation in the SH2 domain, in the presence of vSrc-dsRed in HeLa T-REx FRT cells (Figure 1C). Compared to the unstimulated cells in Figure 1B, the constitutively active tyrosine kinase vSrc-dsRed caused the cytoplasmic localization of STAT5A-eYFP (upper panel, n/c ratio: 0.7, SD: 0.2, n = 30, p < 0.0001) but not STAT5A^R618Q^-eYFP (middle panel, n/c ratio: 1.1, SD: 0.3, n = 30, p = 0.2018) (Figure 1C + D). STAT3-eYFP has been reported to localize to the nuclear compartment under these conditions and served as a positive control in this experiment (lower panel, n/c ratio: 4.4, SD: 1.5, n = 30, p < 0.0001) (Figure 1C + D) [20].

To study the interaction of STAT5A and Src kinases, we performed co-immunoprecipitation (Co-IP) experiments in HeLa T-REx FRT cells. Co-expression of vSrc-dsRed and STAT5A-eYFP resulted in a robust interaction of both proteins and subsequent phosphorylation of STAT5A^Y694^ by vSrc-dsRed (Figure 1E) which is in good agreement with previously published data showing that STAT5A is a direct substrate of Src kinases [21]. However, STAT5A-eYFP appeared to be predominantly cytoplasmic under these experimental settings (Figure 1C, upper right panel). Co-precipitation of vSrc-dsRed with STAT5A^R618Q^-eYFP was decreased compared to STAT5A-eYFP, indicating that the SH2 domain participates in the interaction between STAT5A and Src kinases. In line with this concept, the phosphorylation of Y694 of STAT5A^R618Q^-eYFP was drastically decreased in vSrc-dsRed expressing cells (Figure 1E). Since vSrc is a constitutively active tyrosine kinase lacking negative feedback regulation, we additionally examined the interaction between STAT5A and Hck. Co-IP experiments revealed that Hck-dsRed interacted much stronger with STAT5A-eYFP compared to STAT5A^R618Q^-eYFP. Consistently, the Hck mediated tyrosine phosphorylation of STAT5A^Y694^ was strictly dependent on a functional SH2 domain of STAT5A (Figure 1F) [21]. Therefore, we speculated that STAT5A binds tightly to a phosphorylated tyrosine motif of Src kinases in a SH2 domain dependent fashion and thus fails to localize to the nucleus.

### STAT5A binds to phosphorylated Y416 in the activation loop of Src kinases

**Figure 2:**
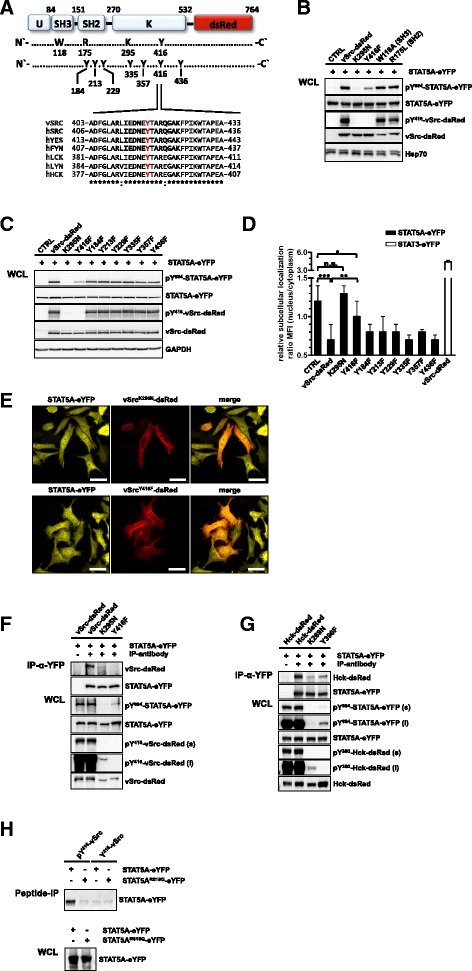
**STAT5A binds to the phosphorylated activation loop of SFK. (A)** Domain structure of vSrc-dsRed. Selected amino acids are highlighted. A multiple sequence alignment of the activation loop of SFK is shown. Autophosphorylation site is highlighted (red). (*) conserved amino acids, (:) similar properties [51]. Bold characters highlight peptide sequence used for precipitation. **(B + C)** HeLa T-REx FRT cells stably expressing STAT5A-eYFP were transfected with the indicated vSrc-dsRed variants. Phosphorylation was analyzed 24 h after transfection using antibodies against pY^416^-Src, Src, pY^694/699^-STAT5A/B and STAT5A. CTRL = untransfected cells. **(D)** Quantification of relative subcellular distribution of STAT5A in HeLa T-REx FRT stably expressing STAT5A-eYFP and the indicated vSrc-dsRed mutants. Mean fluorescence intensity (MFI) of eYFP-fluorescence in the cytoplasm and nucleus were determined using the Zen 2012 software and changes in the ratio between the compartments were plotted. Data show means ± SD of n = 10 cells and were statistically evaluated by Student’s *t*-test. ***p < 0.0005, **p < 0.005, *p < 0.05, n.s. = not significant. **(E)** HeLa T-REx FRT cells stably expressing STAT5A-eYFP were transfected with vSrc^K295N^-dsRed or vSrc^Y416F^-dsRed. Subcellular distribution of STAT5A-eYFP was analyzed 24 h after transfection by confocal microscopy. Scale bars: 20 μm. **(F + G)** HeLa T-REx FRT cells were co-transfected with plasmids coding for vSrc-dsRed (Hck-dsRed), vSrc^K295N^-dsRed (Hck^K269N^-dsRed) or vSrc^Y416F^-dsRed (Hck^Y390F^-dsRed) and STAT5A-eYFP. STAT5-eYFP was immunoprecipitated from cell lysates using a GFP antibody and analyzed by immunoblotting for the presence of vSrc-dsRed (Hck-dsRed) 24 h after transfection. Expression and phosphorylation of STAT5A and vSrc proteins was analyzed in the WCL using antibodies against pY^416^-Src, Src, Hck, pY^694/699^-STAT5A/B and GFP. (s) short exposure, (l) long exposure. **(H)** HeLa T-REx FRT cells expressing STAT5A-eYFP or STAT5A^R618Q^-eYFP were lysed and incubated with a Src-peptide containing tyrosine- or phosphotyrosine 416. Precipitates and WCL were analyzed by immunoblotting using a GFP-specific antibody.

Having shown that the SH2 domain is involved in the interaction with SFK and is required for STAT5A^Y694^ phosphorylation, we analyzed Src kinase tyrosine to phenylalanine mutants to identify the phosphorylation site that facilitates STAT5A recruitment (Figure 2A).

Co-expression experiments revealed that in contrast to kinase activity affecting mutations such as K295N in the ATP-binding pocket and Y416F in the activation loop, mutations of critical residues in the SH3 (W118A) and SH2 (R175L) domains of vSrc-dsRed had no impact on STAT5A^Y694^ phosphorylation, showing that these domains do not contribute to substrate recognition (Figure 2A + B). Notably, the Y416F mutant of vSrc-dsRed displayed a markedly reduced capability to activate STAT5A, which we attributed to the attenuated kinase activity that has been reported for this construct [22].

We subsequently screened the amino acid sequence of vSrc for tyrosine residues that could serve as potential binding sites for the SH2 domain of STAT5A. Six tyrosine residues were included in the analysis, which are putatively surface exposed in the structure of cSrc (PDB: 2SRC) or have been described to be phosphorylated (Figure 2A) [23,24]. With the exception of kinase activity impairing mutations (K295N, Y416F), we observed equal STAT5A^Y694^ phosphorylation levels independent of the Src kinase mutant used in this experiment (Figure 2C). Accordingly, all tested Y to F mutants of vSrc-dsRed but not kinase-dead vSrc^K295N^-dsRed induced a significant redistribution of pSTAT5A from the nuclear to the cytoplasmic compartment with vSrc^Y416F^-dsRed exhibiting the lowest capacity (n/c ratio: 1.0, SD: 0.2, n = 10, p = 0.0382) compared to non-transfected cells (n/c ratio: 1.2, SD: 0.2, n = 10) (Figure 2D + E).

Based on these results, we concluded that the kinase activity of Src is critical for promoting a constitutive cytoplasmic localization of pSTAT5A and hypothesized that the phosphorylation of Y416 in the activation loop of SFK not only regulates kinase activity, but in addition generates a recruitment site for the STAT5A SH2 domain.

To support this idea, we studied the interaction between STAT5A-eYFP and two kinase activity impairing vSrc-dsRed mutants. These mutants are characterized by either the absence (Y416F) or a drastically reduced (K295N) phosphorylation of Y416 in the activation loop (Figure 2F). While STAT5A-eYFP formed a robust complex with vSrc-dsRed, the interaction with kinase dead vSrc^K295N^-dsRed and vSrc^Y416F^-dsRed was drastically attenuated. However, since the latter construct still caused phosphorylation of STAT5A^Y694^, we concluded that substrate recognition is additionally mediated by an activation loop independent mechanism (Figure 2F). Similar observations were made when we investigated the interaction of STAT5A-eYFP with Hck-dsRed or the corresponding inactivating and attenuating mutations Hck^K269N^-dsRed and Hck^Y390F^-dsRed, respectively (Figure 2G, see long exposure for pY^694^-STAT5A-eYFP).

To further demonstrate that the SH2 domain of STAT5A binds to the phosphorylated activation loop of SFK, peptide precipitation assays were performed using a non-phosphorylated peptide and a tyrosine phosphorylated peptide corresponding to the amino acid sequence of the activation loop of the SFK members cSrc (vSrc), Yes and Fyn (Figure 2A). WCL of HeLa T-REx FRT cells expressing either STAT5A-eYFP or STAT5A^R618Q^-eYFP were incubated with the indicated biotinylated peptides coupled to avidin-agarose beads. Immunoblot analysis of the peptide precipitates revealed that STAT5A-eYFP, but not STAT5A^R618Q^-eYFP could be successfully precipitated with the phosphorylated Src-peptide. Binding to the non-phosphorylated peptide appeared comparable to background levels (Figure 2H).

### Cytoplasmic retention of STAT5A in BCR-ABL positive cells is mediated by the phosphorylated activation loop of SFK

We have previously shown that the SFK mediated cytoplasmic retention of STAT5A is dominant over BCR-ABL driven nuclear accumulation [19]. Here, we investigated the role of the phosphorylated tyrosine residue 416 in the activation loop of SFK for the cytoplasmic localization of STAT5A in BCR-ABL expressing cells. Therefore, HeLa T-REx BCR-ABL cells were generated that express the oncogenic tyrosine kinase upon doxycycline treatment.

**Figure 3:**
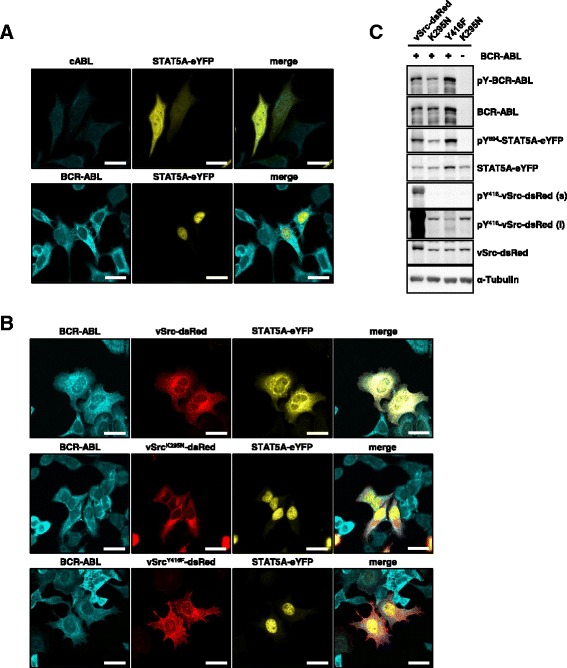
**SFK-mediated cytoplasmic localization of STAT5A is dominant over BCR-ABL induced nuclear accumulation. (A)** HeLa T-REx BCR-ABL cells were transiently transfected with STAT5A-eYFP and either treated with 5 ng/ml doxycycline for 24 h to induce BCR-ABL expression (lower panel) or left untreated (upper panel). Fixation was performed with methanol. Fixed cells were stained for BCR-ABL using a cABL-specific primary antibody and a secondary antibody conjugated to Alexa Fluor-405. The subcellular distribution of STAT5A-eYFP was analyzed by confocal microscopy. Scale bars: 20 μm. **(B)** The subcellular distribution of STAT5A-eYFP was investigated in the presence of vSrc-dsRed (upper panel), vSrc^K295N^-dsRed (middle panel) or vSrc^Y416F^-dsRed (lower panel) in HeLa T-REx BCR-ABL cells that were treated with 5 ng/ml doxycycline for 24 h. Fixation was performed with methanol. Fixed cells were stained for BCR-ABL using an Abl-specific primary antibody and a secondary antibody conjugated to Alexa Fluor-405. Scale bars: 20 μm. **(C)** HeLa T-REx BCR-ABL cells were co-transfected with vSrc-dsRed, or the respective kinase activity affecting mutants vSrc^K295N^-dsRed or vSrc^Y416F^-dsRed and STAT5A-eYFP. The cells were either treated with 5 ng/ml doxycycline for 24 h (lanes 1–3) to induce the expression of BCR-ABL or left untreated (lane 4). Protein expression and phosphorylation in the cellular extracts was investigated by immunoblotting with antibodies against pY^412^-cABL, cABL, pY^694/699^-STAT5A/B, STAT5A, pY^416^-Src and Src. α-Tubulin served as a loading control.

In untreated cells STAT5A-eYFP appeared equally distributed (Figure 3A, upper panel), while the expression of BCR-ABL resulted in the nuclear accumulation of STAT5A-eYFP (Figure 3A, lower panel). In support of our previous results, STAT5A-eYFP failed to accumulate in the nuclear compartment of BCR-ABL positive cells co-expressing vSrc-dsRed (Figure 3B, upper panel). Interestingly, neither kinase dead vSrc^K295N^-dsRed (middle panel) nor vSrc^Y416F^-dsRed (lower panel) was capable of preventing the nuclear accumulation of STAT5A-eYFP in these cells. Consistently, the co-expression of BCR-ABL and kinase dead vSrc^K295N^-dsRed did not alter the phosphorylation status of Y416, demonstrating that the activation loop of Src kinases is not phosphorylated by BCR-ABL (Figure 3C).

Taken together these results reveal that the cytoplasmic retention of pSTAT5A is primarily a consequence of the kinase-substrate interaction, mediated through binding of the phosphotyrosine residue in the activation loop of SFK to the SH2 domain of STAT5A.

### Cytoplasmic recruitment of STAT5A by activated SFK is associated with defective dimerization

Our results so far indicated that the SH2 domain of STAT5A binds to the phosphorylated Y416 in the activation loop of Src kinases. This interaction ultimately interferes with STAT5A nuclear accumulation. Hence, we wondered whether the interaction also impairs STAT5A dimerization.

**Figure 4:**
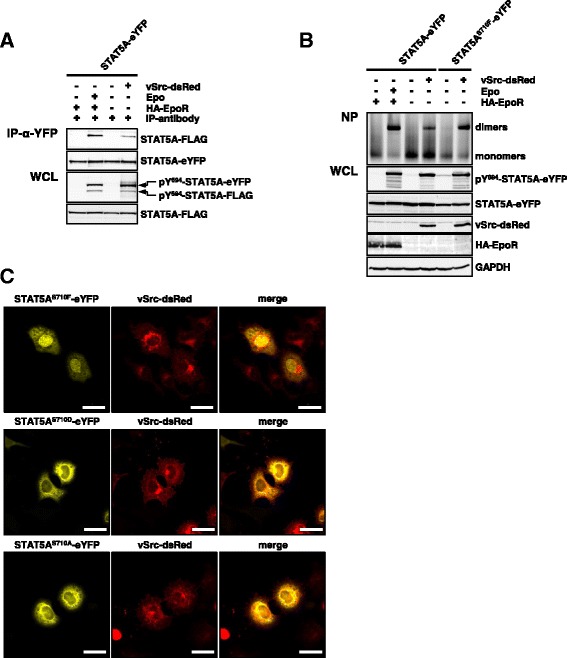
**Binding of STAT5A to Src kinases interferes with dimerization. (A)** HeLa T-REx HA-EpoR cells stably expressing STAT5A-eYFP were transfected with STAT5A-FLAG. The cells were treated with 5 ng/ml doxyxcyline for 24 h to induce the expression of the HA-tagged EpoR and stimulated with 5 U/ml Epo for 30 minutes or left untreated. HeLa T-REx vSrc-dsRed cells stably expressing STAT5A-eYFP were transfected with STAT5A-FLAG. The expression of vSrc-dsRed was induced for 8 h with 5 ng/ml doxycycline or the cells were left untreated. STAT5A-eYFP was immunoprecipitated from cell lysates using a GFP antibody and analyzed by immunoblotting for the presence of STAT5A-FLAG. The expression and phosphorylation of STAT5A-eYFP and STAT5A-FLAG was analyzed in the WCL using antibodies against pY^694/699^-STAT5A/B, GFP and the FLAG-tag. **(B)** HeLa T-REx HA-EpoR cells stably expressing STAT5A-eYFP were treated with 5 ng/ml doxyxcyline for 24 h to induce the expression of the human EpoR and stimulated with 5 U/ml Epo for 30 minutes or left untreated. HeLa T-REx vSrc-dsRed cells stably expressing STAT5A-eYFP or a STAT5A^S710F^-eYFP were treated with 5 ng/ml doxyxcline for 8 h or the cells were left untreated. Cellular extracts were prepared under native conditions and STAT5A-eYFP dimers were separated from monomers by blue native PAGE electrophoresis (NP). STAT5A-eYFP dimer complexes were measured by the detection of the eYFP fluorescence. The cellular extracts were subjected to immunoblotting using antibodies against pY^694/699^-STAT5A/B, STAT5A, Src and the HA-tag of the EpoR. **(C)** Confocal microscopy analysis of HeLa T-REx FRT cells co-expressing vSrc-dsRed together with STAT5A^S710F^-eYFP (upper panel), a serine phosphorylation mimicking mutant STAT5A^S710D^-eYFP (middle panel) or a serine phosphorylation deficient mutant STAT5A^S710A^-eYFP (lower panel). Methanol fixation was performed 24 h after transfection. Scale bars: 20 μm.

In order to compare STAT5A dimerization downstream of erythropoietin receptors (EpoR) and Src kinases, we expressed STAT5A-FLAG in doxycycline treated HeLa T-REx HA-EpoR and HeLa T-REx vSrc-dsRed cells, which were stably transfected with STAT5A-eYFP. EpoR expressing cells were either treated with Epo to induce STAT5A activation or were left untreated (Figure 4A, lanes 1–2). Co-IP experiments revealed that STAT5A-FLAG and STAT5A-eYFP formed dimers in stimulated cells. We did not observe the formation of non-phosphorylated preformed dimers, which has been described for STAT3 [25-27]. Surprisingly, Src mediated phosphorylation of STAT5A resulted in a reduced dimerization compared to Epo stimulation, which could not be attributed to expression or phosphorylation levels of STAT5A (Figure 4A, lanes 2 + 4).

To support these findings we repeated the previous experiment under native conditions. Dimers were separated from monomers using blue native polyacrylamide gel electrophoresis and only fluorescent proteins were detected by fluorescence scanning of the gel. Dimerization of STAT5A-eYFP was efficiently induced by Epo stimulation and accompanied by a simultaneous loss of STAT5A monomers (Figure 4B, lanes 1–2). Subsequent analysis of the corresponding WCL revealed that STAT5A was phosphorylated at tyrosine 694 in response to Epo. In order to compare Epo-induced versus Src kinase-induced dimerization of STAT5A-eYFP, the transcription factor was stably expressed in doxycycline treated HeLa T-REx vSrc-dsRed cells. Interestingly, STAT5A-eYFP dimerization was drastically decreased in response to vSrc-dsRed expression compared to Epo stimulated cells, while STAT5A expression and phosphorylation levels remained comparable (Figure 4B, lanes 2 + 4). Moreover, we were able to rescue Src mediated STAT5A dimerization by using a constitutively active STAT5A variant harboring a serine to phenylalanine mutation in the TA domain at position 710 (Figure 4B, lane 6) [28]. The mechanism rendering STAT5A^S710F^ constitutively active remains unclear but since the observed effect on STAT5A^S710F^ dimerization could not be attributed to increased expression or phosphorylation levels of this mutant, we speculate that F710 stabilizes the STAT5A dimer and thus SFK induced STAT5A^S710F^ homodimers accumulate in the nucleus over time (Figure 4B, lanes 4 + 6).

These results are substantiated by the fact that STAT5A^S710F^-eYFP escaped from the cytoplasmic retention and predominantly localized to the nucleus of HeLa T-REx FRT cells expressing vSrc-dsRed (Figure 4C, upper panel). To demonstrate that dimer stabilization in STAT5A^S710F^ critically depends on F710 and is not a consequence of lacking S710 phosphorylation, this residue was either mutated to aspartic acid (STAT5A^S710D^) to mimic a constitutive serine phosphorylation or to alanine (STAT5A^S710A^) to prevent serine phosphorylation. The subcellular distribution of these mutants was analyzed in the presence of vSrc-dsRed in HeLa T-REx FRT cells by confocal microscopy. In contrast to STAT5A^S710F^-eYFP, STAT5A^S710D^-eYFP and STAT5A^S710A^-eYFP predominantly resided in the cytoplasm of vSrc-dsRed expressing cells, suggesting that the hydrophobic properties of phenylalanine at this position are of importance for the dimer stabilization of STAT5A^S710F^.

**Figure 5:**
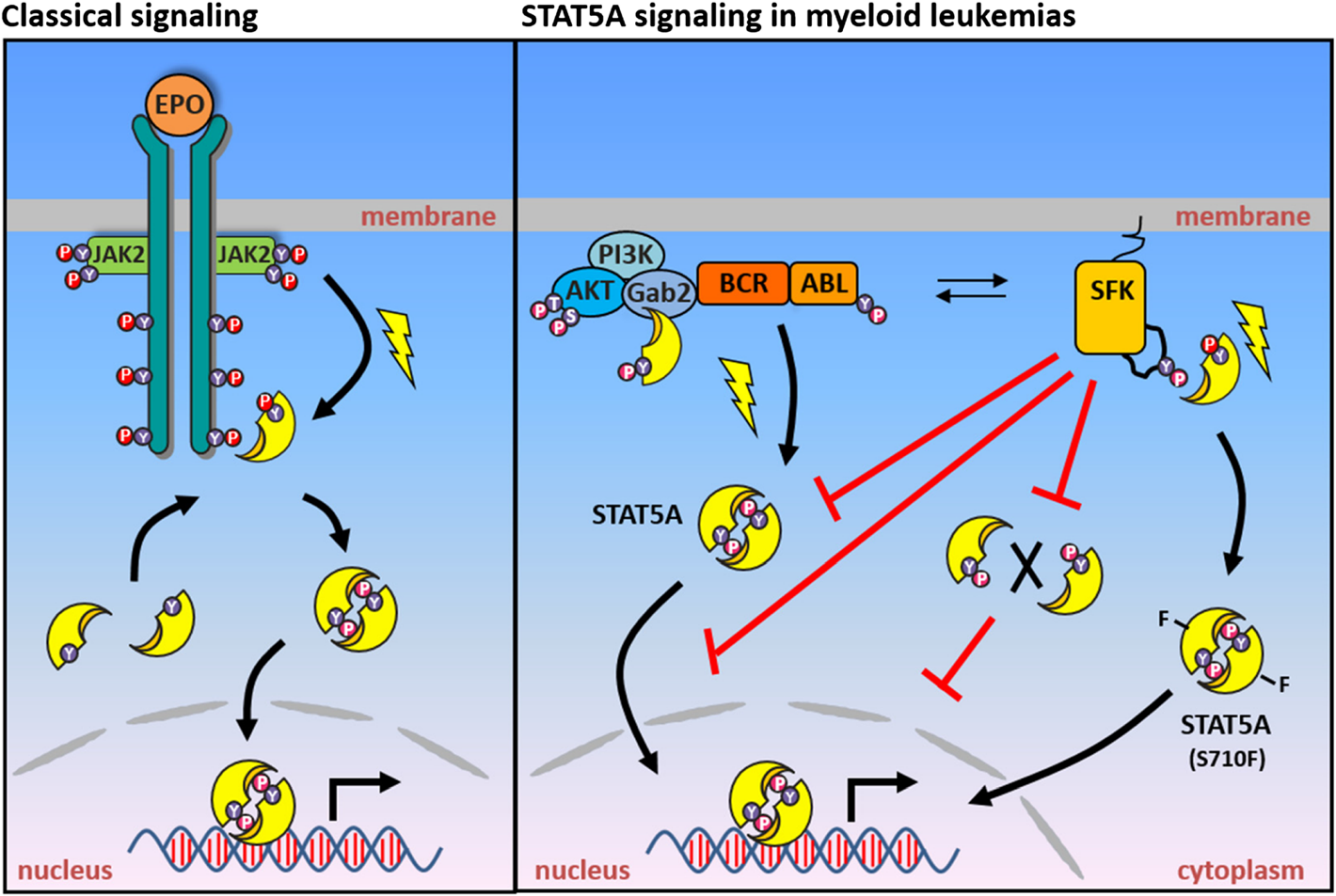
**Activated SFK interfere with dimerization and nuclear translocation of pSTAT5A in BCR-ABL expressing cells.** Left scheme: Classical activation of the JAK2-STAT5A signaling pathway downstream of the EpoR. Right scheme: BCR-ABL directly phosphorylates STAT5A^Y694^ resulting in STAT5A dimerization, nuclear accumulation and finally target gene expression [10]. In the presence of BCR-ABL, a predominantly cytoplasmic localization of pSTAT5A is achieved (i) upon binding to the scaffolding adaptor Gab2 resulting in pro-survival signaling through PI3K/Akt activation [35] and (ii) through binding of the STAT5A SH2 domain to the phosphorylated activation loop of SFK, a mechanism that interferes with STAT5A dimerization and subsequent nuclear accumulation. Constitutively active STAT5A^S710F^ escapes the SFK-mediated cytoplasmic retention. Flashes indicate phosphorylation events.

Conclusively, our results indicate that binding of the STAT5A SH2 domain to the phosphorylated activation loop of SFK leads to a redistribution of the activated transcription factor from the nucleus to the cytoplasm, which is accompanied by impaired STAT5A dimer formation (Figure 5).

## Discussion

STAT5 signaling is implicated in proliferation, survival and myeloid differentiation of hematopoietic cells. Aberrant STAT5 activity is linked to the expression of dysregulated upstream tyrosine kinases such as BCR-ABL, JAK2^V617F^, FLT3-ITD or cKIT^D816V^ and represents a hallmark in a variety of hematological malignancies including myeloproliferative neoplasias (MPNs) [29].

In CML, STAT5 represents a key signaling node since pSTAT5 is essential for the proliferation and survival of leukemic cells and contributes to both the initial transformation and maintenance of the disease [11]. In cytokine induced STAT5 signaling, phosphorylation of a single tyrosine residue leads to dimerization and nuclear accumulation of the transcription factor, which results in DNA binding and gene induction. However, previous reports demonstrated that pSTAT5 predominantly localizes to the cytoplasm of primary BCR-ABL positive CML cells and cKIT^D816V^ positive neoplastic mast cells [13,19,30]. Growing evidence supports the idea that cytoplasmic pSTAT5 cooperates with the PI3-K/Akt signaling pathway through the association with the scaffolding adaptor Gab2 in order to maintain prosurvival signaling [13,31-35].

Most recently, we identified Src family kinases (SFK) to be involved in mediating the cytoplasmic retention of pSTAT5A in BCR-ABL expressing cells, a process that involves the SH2 domain of STAT5A. As a consequence, STAT5A target gene expression was shown to be attenuated, which could be rescued through the specific inhibition of SFK [19]. Furthermore, it has been shown that Src kinases phosphorylate STAT5A in *in-vitro* kinase assays, providing strong evidence for a direct interaction, which is further substantiated by the co-localization of pSTAT5 with constitutively active Hck in podosomes [21,36].

However, the role of the STAT5A SH2 domain in this context is still unresolved. In order to clarify the mechanism underlying the Src kinase mediated cytoplasmic retention of STAT5A, we co-expressed STAT5A-eYFP or STAT5A^R618Q^-eYFP with the SFK members Hck-dsRed and vSrc-dsRed. We confirmed the observation that the SH2 domain of STAT5A is involved in the formation of a stable complex with both SFK, which contributes to the cytoplasmic localization of pSTAT5A. Moreover, phosphorylation of STAT5A^Y694^ by SFK requires an intact STAT5A SH2 domain, which supports the idea of an exceptional interaction between the kinase and its substrate. Interestingly, the inactivating mutation R618Q in the SH2 domain of STAT5A did not result in a complete loss in binding to SFK, which indicates that multiple domains contribute to the interaction.

In line with this concept, the SFK mediated activation of the STAT family members STAT3 and STAT5B was shown to be largely independent of a functional SH2 domain (data not shown) [19]. Consistently, nuclear functions of STAT3 and STAT5B were reported to be important for vSrc mediated cellular transformation [37-40].

Furthermore, the specific knockdown of STAT5B, but not STAT5A, was shown to be associated with a loss of CML cell proliferation. In the context of BCR-ABL signaling, stress protection through the regulation of reactive oxygen species could be attributed to STAT5A functions independent of its transcriptional activity, suggesting a cytoplasmic role of pSTAT5A in this context [41]. In contrast, other studies postulated a requirement of the transcriptional activity of STAT5A for the regulation of ROS, pointing towards a more nuclear function of STAT5A in CML cells [42,43].

In order to further characterize the SFK/STAT5A protein interaction and its contribution to the cytoplasmic localization of pSTAT5A, tyrosine to phenylalanine mutations were introduced into vSrc-dsRed. Out of seven Y to F mutations only the expression of vSrc^Y416F^-dsRed, which lacks the phosphorylation site in the activation loop, resulted in a decreased STAT5A^Y694^ phosphorylation. This observation is not surprising, since the Y416F mutation negatively affects kinase activity. However, subsequent interaction studies revealed that binding of STAT5A to vSrc^Y416F^-dsRed and vSrc^K295N^-dsRed is significantly reduced compared to vSrc-dsRed, which correlates with a substantial loss of Y416 phosphorylation and a decreased capacity to induce the cytoplasmic localization of STAT5A. In addition, STAT5A could be successfully precipitated with a phosphorylated peptide corresponding to the sequence of the activation loop of SFK in a SH2 domain dependent fashion. However, our experiments also demonstrate that the binding of STAT5A to SFK is not limited to phosphotyrosine-SH2 domain interactions, which has also been shown for STAT5/Hck complexes in BCR-ABL transformed haematopoietic cells and TEL-ABL expressing Ba/F3 cells [18,44].

Accordingly, our findings suggest that phosphorylation of the activation loop, which is drastically reduced in kinase dead vSrc^K295N^ and absent in vSrc^Y416F^, is required for the SFK induced cytoplasmic localization of STAT5A in the presence of BCR-ABL. Taken into account that the SFK members Hck and Lyn are typically expressed in myeloid cells and are constitutively activated by the p210 isoform of BCR-ABL, it is tempting to speculate that activated SFK contribute to the persistent cytoplasmic localization of pSTAT5A observed in primary CD34^+^ CML cells [13,17,19,45].

Having shown that SFK/STAT5A protein complexes are stabilized by a phosphotyrosine-SH2 domain mediated interaction, which is accompanied by a lack of nuclear accumulation, we wondered whether this interaction affects STAT5A dimerization. Native gels revealed that the dimer formation of STAT5A in response to activation through SFK is significantly reduced compared to Epo stimulation, despite equal phosphorylation levels. Interestingly, our unpublished data suggest that in response to SFK, STAT5A is also phosphorylated at residues different from Y694, which has also been reported for STAT5B [46]. Taken into account that a cytoplasmic crosstalk between STAT5 and p85, the regulatory subunit of PI3-K, has been postulated, phosphorylated STAT5A in complex with SFK might serve as a cytoplasmic scaffold for SH2 domain containing proteins involved in this crosstalk [34,35].

The role of constitutive STAT5 activation and its contribution to the development of blood malignancies has been intensively investigated by the use of STAT5 activating mutations. A single point mutation in the transactivation domain (S710F) renders STAT5A constitutively active and promotes factor independent growth of Ba/F3 cells [28]. Despite the capability of STAT5A^S710F^ but also STAT5A^S710A^ to rescue STAT5A^−/−^ T cell proliferation, only STAT5A^S710F^ was shown to result in the induction of leukemia upon expression in the bone marrow of wild-type and STAT5^−/−^ mice, demonstrating that the hydrophobic properties of phenylalanine are required for the oncogenic potential of STAT5A^S710F^. However, a detailed understanding of the mechanism rendering STAT5A^S710F^ constitutively active is lacking.

SFK failed to interfere with the dimerization of STAT5A^S710F^-eYFP, which is in line with the observation that STAT5A^S710F^-eYFP escapes from the SFK mediated cytoplasmic retention and predominantly localizes to the nuclear compartment. Phosphorylation of S710 is most likely not involved in the regulation of the subcellular distribution of STAT5A, since mutants designed to impair (STAT5A^S710A^) or to mimic (STAT5A^S710D^) serine phosphorylation could not rescue the SFK mediated block in nuclear accumulation of STAT5A. Conceivably, our data suggest that the oncogenic properties of STAT5A^S710F^ are a consequence of increased dimer stability that either interferes with the deacetylation/sumoylation cycle, which is involved in STAT5 inactivation, or impairs the recruitment of STAT5 phosphatases such as SHP-2 and PTP1B [47-49].

## Conclusions

Our findings identify Y416 in the conserved activation loop of SFK as a recruitment site for the SH2 domain of STAT5A. The phosphotyrosine-SH2 domain interaction is required for the activation of STAT5A. At the same time, this interaction promotes the cytoplasmic localization of the transcription factor in BCR-ABL^+^ cells, which is accompanied by an impaired dimerization. The oncogenic STAT5AS710F mutant escapes from cytoplasmic retention through SFK as a consequence of increased dimer stability. These results reveal important structural aspects of the cytoplasmic localization of pSTAT5A in BCR-ABL expressing cells and will contribute to our understanding of STAT5A mediated cytoplasmic signaling.

## Methods

### Hormones and antibodies

Erythropoietin (Roche, Basel, Switzerland) was used at a concentration of 1 or 5 U/ml. Anti-pY^694/699^-STAT5A/B (#9351), anti-pY^416^-Src (#2101), anti-pY^412^-Abl (#2865), anti-Hsp70 (#4872, Cell Signaling, Beverly, USA), anti-STAT5A (clone #5073, rabbit polyclonal antiserum was kindly provided by Richard Moriggl, Ludwig Boltzmann Institute for Cancer Research (LBI-CR), Vienna, Austria), anti-GFP (600-103-215, Rockland, Gilbertsville, USA), anti-FLAG (F3165), anti-α-tubulin (T5168, Sigma, St. Louis, USA), anti-GAPDH (sc-32233), anti-Abl (sc-131), anti-Hck (sc-72), anti-cSrc (sc-19, Santa Cruz Biotechnology, Santa Cruz, USA), anti-vSrc (MABS193, Millipore, Billerica, MA, USA) and anti-HA (MMS-101R, Covance, Princeton, New Jersey, USA) antibodies were used for immunoblotting. The anti-pY^416^-Src antibody was used to detect phosphorylated Y390 of Hck. Anti-rabbit, anti-mouse and anti-goat antibodies conjugated to horseradish peroxidase (HRP) were ordered from DAKO (Hamburg, Germany).

### Plasmid constructs

The cDNA of murine STAT5A was either cloned into the pcDNA3.1, pcDNA5/FRT/TO (Invitrogen, Paisley, UK) or pLentiLox (Vector Core, University of Michigan, USA) expression vectors and fused to the cDNA of the enhanced yellow fluorescent protein (eYFP) or the FLAG-tag (N’-DYKDDDDK-C’). The cDNA of vSrc and human Hck (kindly provided by Isabelle Maridonneau-Parini, Université Paul Sabatier, Toulouse, France) was fused to monomeric dsRed and cloned into pcDNA3.1 or pcDNA5/FRT/TO expression vectors. The cDNA of BCR-ABL (p210, kindly provided by Tim Brümmendorf, Medizinische Klinik IV, RWTH Aachen, Germany) was cloned into the pcDNA5/FRT/TO expression vector. The cDNA of the human erythropoietin receptor (EpoR) was cloned into the pcDNA5/FRT/TO expression vector and fused to the cDNA of the hemagglutinin tag (HA). Fluorescent proteins and the FLAG-tag were fused to the C-terminus of target proteins, whereas the HA-tag was fused to the N-terminus of the human EpoR. Point mutants of STAT5A, Hck and vSrc were generated with the PCR based QuikChange XL Site-Directed Mutagenesis Kit according to manufacturer’s instructions (Agilent Technologies, Santa Clara, CA, USA).

### Cell culture, transfection and inducible cell lines

HeLa T-REx (Invitrogen, Paisley, UK) cells were grown in Dulbecco’s modified Eagle’s medium (DMEM)/GlutaMAX (Gibco, Paisley, UK). Transient transfection of HeLa T-REx cells was performed using *Trans*IT®-LT1 (Mirus, Madison, USA) according to manufacturer’s instructions. Stable transfection of HeLa T-REx cells for the inducible expression of BCR-ABL, HA-EpoR, vSrc-dsRed and Hck-dsRed were generated with the Flp-In system (Invitrogen, Paisley, UK) using 250 μg/ml hygromycin B (PAA, Austria) and 15 μg/ml blasticidin (Invivogen, Toulouse, France) for selection. Non-inducible HeLa T-REx FRT cells were maintained in DMEM medium supplemented with 200 μg/ml Zeocin (Invivogen, Toulouse, France) and 15 μg/ml blasticidin. The expression of the gene of interest was induced with 5 ng/ml doxycycline (Sigma, St. Louis, USA) for 24 h or as indicated. All media were supplemented with 10% FCS (Lonza, Verviers, Belgium) and 25 U/ml penicillin/streptomycin (Lonza, Verviers, Belgium). Cells were incubated at 37°C in a water-saturated atmosphere with 5% CO_2_.

### Lentiviral mediated stable transduction

To achieve stable and constitutive expression of STAT5A constructs in inducible HeLa T-REx cells, self-inactivating lentiviral constructs were used according to the guidelines of the RNAi consortium. Briefly, the cDNA of STAT5A-eYFP and STAT5A^S710F^-eYFP were cloned into the lentiviral pLentiLox expression vector downstream of the CMV promoter (Vector Core, University of Michigan, USA). HEK293-T packaging cells were seeded on 6-cm dishes on the first day to be 80-90% confluent at the time of transfection. On the second day, HEK293-T cells were transiently co-transfected with the lentiviral vector, psPAX2 (packaging plasmid) and pMD2.G (envelope plasmid). Eighteen hours after transfection the culture medium was replaced by high serum growth medium containing 30% FCS. Viruses were harvested 24 and 48 hours after medium exchange. For the infection of target cells, virus-containing supernatant was supplemented with 8 μg/ml polybrene (Sigma, St. Louis, USA) prior to incubation. For sorting by fluorescence-activated cell sorter analysis (FACS), cells were grown for 10 days and sorted by eYFP gating.

### Preparation of cell lysates, SDS-PAGE and immunoblotting

HeLa T-REx cells were washed with PBS (137 mM NaCl, 2.5 mM KCl, 8 mM Na_2_HPO_4_, 1.5 mM KH_2_PO_4_, adjusted to pH 7.4) once and lysed with RIPA lysis buffer (50 mM Tris–HCl, pH 7.4, 150 mM NaCl, 1 mM EDTA, 0,5% Nonidet P-40, 1 mM NaF, 15% glycerol) supplemented with phosphatase/protease inhibitors (1 mM Na_3_VO_4_, 0.5 mM EDTA, 0.25 mM phenylmethylsulfonylfluoride (PMSF), 5 μg/ml aprotinin, 2.5 μg/ml leupeptin) according to standard procedures. The proteins were separated by SDS-PAGE and transferred to a PVDF membrane with subsequent immunodectection using specific antibodies. Primary and HRP-conjugated secondary antibodies were diluted in TBS-N buffer (20 mM Tris–HCl, pH 7.5, 135 mM NaCl, 0.1% Nonidet P-40). Membrane-bound antibody complexes were detected by chemiluminescence (ECL, Millipore, Billerica, MA, USA).

### Blue Native PAGE and detection of enhanced YFP

Dimerization of fluorescently eYFP-labeled STAT5A constructs was analyzed in HeLa T-REx vSrc-dsRed and HeLa T-REx HA-EpoR cells. Cell lysis was performed under native conditions using the lysis buffer of the NativePAGE™ Sample Prep Kit (Invitrogen, Paisley, UK) supplemented with 2% digitonin (Sigma, St. Louis, USA). The lysates were cleared by centrifugation and incubated with Coomassie brilliant blue G-250 and separated over night at 4°C using the NativePAGE™ Bis-Tris gel system with a gradient polyacrylamide gel (4-16%) according to manufacturer’s instructions. Dimerization of STAT5A-eYFP was analyzed by the detection of the eYFP fluorescence with a typhoon fluorescence scanner (GE Healthcare, Germany) by excitation with a 488 nm laser line. The emission was detected using a 515–555 nm bandpass filter.

### Peptide precipitation assay

HeLa T-REx FRT cells expressing STAT5A-eYFP or STAT5A^R618Q^-eYFP were lysed with storage buffer (150 mM NaCl, 50 mM Tris–HCl, pH 7.5, 0.1 mM EDTA, 10% glycerol, 0.5% Nonidet P-40) supplemented with phosphatase/protease inhibitors. Lysates were cleared by ultracentrifugation (100.000 g, 30′, 4°C). Biotinylated peptides corresponding to the activation loop sequence of Src family kinases (N’-RLIEDNE(p)YTARQGAK-C’) were synthesized with >95% purity (Genosphere Biotechnologies, Paris, France). 25 nmol of the peptides were coupled to NeutrAvidin Agarose Resin (Thermo Scientific, Pittsburgh, PA, USA) for 2 h at 4°C, washed twice with storage buffer and incubated with 1 mg of the lysate over night at 4°C. Peptide/protein complexes were washed 3 times with storage buffer for 10 minutes and the precipitates were analyzed by SDS-PAGE and immunoblotting.

### Immunoprecipitation

HeLa T-REx cells were lysed with RIPA lysis buffer (50 mM Tris–HCl, pH 7.4, 150 mM NaCl, 1 mM EDTA, 0,5% Nonidet P-40, 1 mM NaF, 15% glycerol) supplemented with phosphatase/protease inhibitors and the lysates were cleared by ultracentrifugation (100.000 g, 30′, 4°C). Protein G Sepharose (GE Healthcare, Dornstadt, Germany) was coupled to 1 μg anti-GFP antibody for 16 h at 4°C, washed twice with RIPA buffer and subsequently incubated with 1 mg of lysates over night at 4°C. Antibody/protein complexes were washed 3 times with RIPA buffer for 10 minutes and precipitates were analyzed by SDS-PAGE and immunoblotting.

### Indirect immunofluorescence and confocal microscopy

HeLa T-REx cells expressing fluorescently labeled fusion proteins were grown on glass cover slips. Methanol fixation was performed at room temperature as has been described previously [50]. Primary antibodies were diluted 1:100, secondary Alexa Fluor-405 conjugated antibodies (Invitrogen, Paisley, UK) were diluted 1:1000 and applied for 45 minutes. The cover slips were mounted with ImmuMount (Thermo Scientific, Pittsburgh, PA). Fluorescence images of adherent cells were generated with a Zeiss LSM 710 confocal microscope (Zeiss, Jena, Germany), using the Zeiss LD C-apochromat 40x/1.1 water objective. Images represent confocal slices of approximately 1 μm and were analyzed with the ZEN 2012 software (Zeiss, Jena, Germany).

### Quantification of the subcellular distribution of STAT5A

HeLa T-REx cells expressing STAT3-eYFP and STAT5A-eYFP fusion constructs were fixed as described previously. Tilescan images (9 images, zoom 0.6) of adherent cells were generated with a Zeiss LSM 710 confocal microscope (Zeiss, Jena, Germany), using the Zeiss LD C-apochromat 40x/1.1 water objective. Tilescan images represent confocal slices of approximately 1 μm. Mean fluorescent intensities (MFI) of the YFP fluorescence in the cytoplasm and nucleus were calculated for double transfected cells using the profile function of the ZEN 2012 software (Zeiss, Jena, Germany). Relative ratios of nuclear versus cytoplasmic fusion proteins were plotted using Graph Pad Prism 4 software and statistically evaluated by Student’s *t*-test.

## Abbreviations

BCR-ABL: Breakpoint cluster region – Abelson kinase
IP: Immunoprecipitation
N’: N-terminus
C’: C-terminus
HA: Hemagglutinin
FLT3-ITD: Fms-like tyrosine kinase 3 – internal tandem duplication
WCL: Whole cellular lysate
